# Parallel activation of multiple languages in trilinguals: Evidence from eye movements and individual differences

**DOI:** 10.1017/S0142716426100733

**Published:** 2026-07-13

**Authors:** Clara Fridman, Natalia Meir, Viorica Marian

**Affiliations:** 1 Department of Communication Sciences and Disorders, Northwestern University, Evanston, USA; 2 Department of English Literature and Linguistics, Bar-Ilan University, Israel; 3 Bar-llan University Leslie and Susan Gonda Multidisciplinary Brain Research Center, Israel

**Keywords:** Co-activation, eye-tracking, individual differences, trilingual

## Abstract

Trilinguals’ three languages can be activated simultaneously, but the patterns of co-activation in the language network remain unclear. Our study examines language processing and co-activation in trilingual speakers of Russian, Hebrew, and English. Participants completed a visual search eye-tracking experiment in three languages with cross-linguistic competitors from either one or two languages present in the visual display, as well as vocabulary assessments indexing proficiency and a Flanker task indexing cognitive control. When competitors were present from only one non-target language, higher proficiency in the non-target language increased co-activation, while higher proficiency in the target language decreased co-activation or showed no effects. When competitors were present from two non-target languages, higher proficiency decreased co-activation of one competing language and had a variable effect on the second. Decreased co-activation of more-dominant languages and increased co-activation of less-dominant languages was associated with stronger cognitive control. We conclude that trilinguals co-activate languages similarly to bilinguals when competitors from only one language are present but show different patterns when competitors are present from two languages, with individual differences in language proficiency, dominance, and cognitive control shaping levels of co-activation of each language in the trilingual cognitive system.

Imagine a Russian–Hebrew–English trilingual hearing the Russian word *karta* (map). As she hears the first syllable *kar*, her brain activates similar-sounding lexical candidates in all of her languages, which compete for selection (see Figure 1). She may first activate the Hebrew word *karit* (pillow), which is later ruled out as more phonemes unfold. However, since she also speaks English, hearing *kar* might additionally activate the English word *carpet*, adding another layer of competition. In multilingual co-activation, the target word needs to be identified while filtering out lexical competitors in several languages. The ability to manage cross-linguistic co-activation can vary depending on variables such as proficiency in each language, relative language dominance, or cognitive factors. For example, if the trilingual’s English proficiency is low, she may activate *carpet* much less than *karit.* However, if her Russian, the target language in this case, is less dominant, both Hebrew and English competitors could be activated to a similar extent. Trilingual language processing thus becomes increasingly complex and susceptible to individual differences in language experience and cognitive profiles.

**Figure 1.**
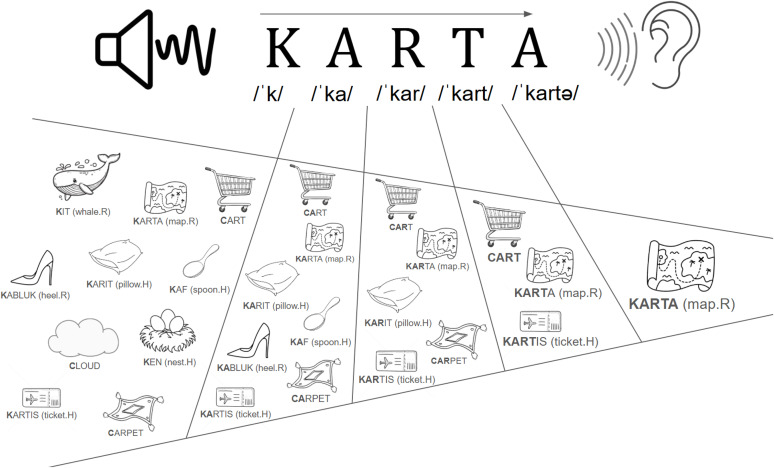
Figure 1 long description. Example of cross-linguistic competition in a Russian–Hebrew–English trilingual. As the Russian target word *karta* (map) unfolds, the trilingual activates lexical candidates in all three languages. With each new phoneme, candidates that no longer fit the auditory input are eliminated until the target word is selected.

Over the past two decades, research has consistently shown that multilinguals’ languages can be co-activated in parallel (for a detailed discussion, see Marian, 2023). Co-activation has been most commonly demonstrated in bilinguals, with recent work extending these findings to trilinguals (Otwinowska, 2024; Pathak et al., 2024). Most research on trilingual speakers has focused on the early stages of L3 acquisition (see Puig-Mayenco, 2024) or on off-line cognate processing (e.g., Lijewska, 2023; Szubko-Sitarek, 2011; van Hell & Dijkstra, 2002). Less is known about online processing in trilinguals (Lemhöfer, 2023; Pereira Soares & Rothman, 2021) and how individual differences in language proficiency and cognitive control may influence trilingual co-activation.

Among bilinguals, higher proficiency in a non-target language has been found to increase cross-linguistic co-activation during processing (Blumenfeld & Marian, 2007; Marian et al., 2013), while higher proficiency in a target language can decrease such co-activation (Berghoff et al., 2021; Sarrett et al., 2022). Meanwhile, the effects of proficiency in trilingual online processing are largely underexplored.

Stronger cognitive control has been linked to better management of cross-linguistic competition (Blumenfeld & Marian, 2011; Mercier et al., 2014). However, the relationship between co-activation and cognitive control has been investigated primarily in bilinguals, and far less is known about how trilinguals manage competition between three languages rather than just two (Chung-Fat-Yim et al., 2023). This is despite the fact that much of the world’s population knows^1^ at least three languages (Bamgbose, 2011; Grosjean, 2013), including over 20% of European adults (Eurostat, 2022).

Given past research on co-activation in bilinguals, language proficiency and cognitive control likely also play a role in trilingual co-activation. For example, the BLINCS (*Bilingual Language Interaction Network for Comprehension of Speech*; Shook & Marian, 2013) model of bilingual processing posits that individual differences in proficiency and cognitive control may influence co-activation strength. Moreover, these effects may be more pronounced and more variable depending on the number of languages activated at a time and on trilinguals’ relative dominance in each of their languages.

In the present study, we implement a visual world eye-tracking paradigm to investigate how trilingual co-activation patterns might vary depending on the particular languages and number of languages activated. We then explore the effects of language proficiency and cognitive control on co-activation, and whether these effects differ when competition comes from one versus two languages at a time. Following calls to study more diverse language combinations and speaker types (Fridman & Özsoy, 2024; Otwinowska, 2024; Puig-Mayenco, 2024), we focus on speakers of three typologically distinct languages. Our participants are heritage language (HL) speakers of Russian, dominant and immersed in their societal language (SL^2^), Hebrew, and proficient in their school-studied third language (L3), English. Through our investigation of individual differences and co-activation in trilinguals, we aim to expand our understanding of how multilinguals process three languages, offering novel insights into the cognitive dynamics that underlie language control and competition across multiple languages.

## Multilingual co-activation in language processing

As a spoken word unfolds, listeners activate multiple potential candidates until the target word is selected (Marslen-Wilson, 1987; McClelland & Elman, 1986). For instance, as one hears the word “letter,” beginning with “le-,” the listener might initially activate several candidate words that overlap at onset, such as “lemon,” “lettuce,” and “letter.” As the input progresses to “lett-,” the activation of “lemon” will decrease in favor of other candidates that better match the available speech signal (e.g., “lettuce”) until a unique phoneme is recognized and selected. This process is referred to as co-activation, wherein listeners must select a target word from a set of phonological competitors.

Competition effects during speech processing have been found for monolinguals within their one language (Spivey-Knowlton et al., 1998), for bivarietal speakers between their language varieties (Keller et al., 2023), and for bilinguals between their two languages (Marian & Spivey, 2003a; Marian & Spivey, 2003b). In fact, the competition process becomes increasingly challenging for bilinguals, who must manage not only similar-sounding words from their L1 but also from their L2, greatly increasing both the potential for activation and the effort needed to control competition. For the example above, imagine that in addition to “lemon,” the first phonemes of the target word also activate Spanish *leche* “milk.” Thus, the potential for cross-linguistic competition grows with each additional language.

To investigate the mechanisms of cross-linguistic competition, researchers often use the visual world paradigm (see Huettig et al., 2011; Mercier et al., 2014; Tanenhaus et al., 1995), wherein participants hear spoken input and select items from a multi-item visual display. Through this paradigm, eye movements to visual items serve as a proxy for attentional deployment, which can reveal lexical access of item labels. The visual world paradigm has provided ample evidence for co-activation, although findings are sometimes asymmetrical. For instance, while some studies find bidirectional cross-linguistic activation during spoken word recognition (Blumenfeld & Marian, 2007; Lagrou et al., 2011; Marian & Spivey, 2003b; McDonald & Kaushanskaya, 2020), others find co-activation of the L1 during L2 processing only (Chambers & Cooke, 2009; Marian & Spivey, 2003a; Shook & Marian, 2016; Weber & Cutler, 2004) and yet others report co-activation of the L2 during L1 processing only (Ju & Luce, 2004; Spivey & Marian, 1999), with differences likely due to language dominance and immersion contexts at the time of testing.

Shook and Marian (2013) suggest that language activation may be further affected by variation in cognitive and language experience factors. Indeed, this suggestion ties together two accounts explaining how multilinguals manage competition from different languages. In one account, competition is handled through a top-down process that globally inhibits competitors from non-target languages (Green, 1998). In contrast, and more likely, activation-based accounts (e.g., BIA+; BLINCS) suggest lexical access is non-selective and competition is resolved as a function of the relative strength of co-activated lexical items rather than the inhibition of an entire language (Dijkstra & van Heuven, 2002; Shook & Marian, 2013). We investigate the effects of both language proficiency and cognitive control on trilingual processing.

## The role of language proficiency in cross-linguistic competition

The activation of a cross-linguistic competitor is most reliably observed when multilinguals are highly proficient in the competitor language (Blumenfeld & Marian, 2007; Marian et al., 2013; Soto & Schmid, 2024). For instance, Blumenfeld and Marian (2013) found that English–Spanish bilinguals with higher Spanish proficiency showed increased co-activation of Spanish competitors during English processing, echoing similar findings from English–German bilinguals in Blumenfeld and Marian (2007).

Some studies have shown that less proficient languages could be co-activated when processing more-dominant ones (Bice & Kroll, 2015; Lee et al., 2018; Marian & Spivey, 2003b), while others found no evidence of co-activating less-dominant languages during more-dominant language processing (e.g., Ju & Luce, 2004; Weber & Cutler, 2004). Duñabeitia et al. (2010) propose that these asymmetrical effects stem from the relative proficiencies between the target and competitor languages, suggesting that balanced and unbalanced bilinguals who process a particular language would show starkly different results, with only the former group demonstrating bidirectional activation.

By contrast, in a recent study of L1-dominant bilinguals, Berghoff and Bylund (2024) found *less* co-activation of the L2 competitor with increased L2 proficiency. The authors explain this finding by positing that, with lower L2 proficiency, L2 lexical representations rely on—and are more closely tied to and therefore more easily co-activated by—L1 representations. However, with increased L2 proficiency, L2 lexical representations become more independent from their L1 counterparts and are easier for the bilingual to inhibit. This explanation from Berghoff and Bylund (2024) reflects previous results from Berghoff et al. (2021), wherein higher proficiency in the target L2 was associated with less co-activation of the L1 competitor. A comparable effect was observed by Sarrett et al. (2022), where L1 co-activation decreased the more proficient a participant was in the target L2. Chambers and Cooke (2009), however, did not find proficiency in target L2 to affect co-activation of the L1 competitor.

In sum, bilingualism research has routinely found effects of proficiency on co-activation, although—like co-activation itself—these effects can sometimes be unidirectional. Furthermore, while some studies report more co-activation with higher proficiency in the competitor language, others report the opposite pattern. Effects of target language proficiency have been less commonly studied, but evidence either shows that increased proficiency in the target language leads to diminished co-activation, or finds null effects; predictably, no studies have found that higher proficiency in the target language leads to greater cross-linguistic co-activation.

Looking to trilinguals, language experience in L1 and L2 has been proposed to affect L3 development (Puig-Mayenco, 2024), and by extension, likely L3 processing, as well. However, specific effects of trilinguals’ proficiency on online processing are understudied, so we must extrapolate based on related work. Using a lexical decision task to study L1 cognate processing, van Hell and Dijkstra (2002) found that trilinguals with high proficiency in L3 showed a facilitation effect, processing L1–L3 cognates faster than non-cognates. However, as a low L3 proficiency group did not produce the same results, these findings suggest that L1 processing may co-activate both less-dominant languages, but only when a certain proficiency threshold is met (van Hell & Tanner, 2012).

A key novelty of the present work is considering proficiency in both the competitor languages and the target language, as we move beyond the binary bilingual paradigm. In the case of unbalanced trilingualism, an individual might be hearing spoken input in the most-dominant, least-dominant, or middle-dominance language, with cross-linguistic competition from 1 to 2 more-dominant or 1 to 2 less-dominant languages, or one more-dominant and one less-dominant language. This increased number of permutations suggests that proficiency in both the target and the competitor languages may jointly influence the extent of co-activation.

## The role of cognitive control in cross-linguistic competition

Another mechanism that contributes to lexical activation and selection is cognitive control, which prioritizes certain pieces of information over others during processing through facilitation and inhibition (Ness et al., 2023). In bilinguals, stronger cognitive control has often been tied to better management of competition during spoken word recognition (Blumenfeld & Marian, 2011). Shook and Marian (2013) propose that, when processing a less-dominant language, overriding competition from the more-dominant language would be more effortful than the reverse scenario. Indeed, multiple studies have shown that bilinguals with stronger cognitive control experience less co-activation (Chen et al., 2017; Giezen et al., 2015; Mercier et al., 2014; but see Prior et al., 2017 for null results).

Interestingly, Blumenfeld and Marian (2013) found stronger cognitive control in bilinguals to correlate with *more* cross-linguistic activation, but only during early stages of word recognition. Their finding supports results from Meuter and Allport (1999), who showed that inhibiting a more-dominant competitor when processing a less-dominant language could lead to increased co-activation during the subsequent processing of a more-dominant language. This effect of reversed language dominance, where more-dominant language activation is expected but a less-dominant language is activated instead, has been widely demonstrated across different language combinations and tasks (Gavino et al., 2025; Goldrick & Gollan, 2023).

Taken together, the studies discussed above suggest that cognitive control may play a role in bilingual processing (Green & Abutalebi, 2013; Ness et al., 2023), and we extrapolate that it may also come into play during trilingual processing. Schroeder and Marian (2017) propose that trilinguals may filter their languages dichotomously as target or non-target, with all non-target languages grouped together regardless of their number. If this were the case, the effects of cognitive control on competitor co-activation would be the same regardless of whether one or two competing languages were activated at a time, and regardless of the languages being activated.

However, in a production task, Linck et al. (2012) investigated the role of cognitive control during language-switching among trilinguals and found an effect only when processing the most dominant language. This finding suggests that cognitive control might impact trilingual co-activation differently depending on individuals’ relative dominance in each language. This suggestion would align with Green and Abutalebi’s (2013) hypothesis that cognitive control may adapt in the face of different demands, which could include differences in language dominance.

## The present study

To our knowledge, the visual world eye-tracking paradigm has thus far only been used to study co-activation in bilingual populations, largely due to the methodological complexities of adding a third language (Lemhöfer, 2023; Chung-Fat-Yim et al., 2023; Pathak et al., 2024). In the present study, we re-analyze data from Fridman and Meir (2025)^3^, the first study to investigate trilingual co-activation with eye-tracking, to pose three novel research questions:

RQ1 **How does the number of competing languages modulate cross-linguistic co-activation in trilinguals?**

We hypothesize that the strength of co-activation may depend on the number of languages activated at a time. Studies on priming have found that a greater number of simultaneous competitors impede processing (Dufour & Peereman, 2003), although it is unclear which competitor would draw the most attention. Contextualizing these findings in the present study, we may expect a greater magnitude of co-activation of one or both languages with two simultaneously activated competitor languages compared to conditions with only one. This would indicate that activating one competitor language, as bilinguals do, or two competitor languages simultaneously, as trilinguals might do, is likely quantitatively different.

In an alternative scenario, when processing two simultaneous competitor languages, co-activation of one or both competitors may decrease. This could happen either due to an imbalance in activation (where one cross-linguistic competitor is activated more than the other) or because the listener reaches a competition threshold and is no longer attuned to additional competitors (Schroeder & Marian, 2017). We may expect this latter pattern to emerge as a function of language dominance, and such a divergence would indicate that simultaneously processing one versus two competitor languages differs qualitatively, as well.

RQ2 **How does the type (heritage, societal, L3) of target and competitor language influence co-activation patterns?**

We assess effects of language type primarily in the context of language dominance. Based on previous findings, we may find stronger co-activation when more-dominant languages compete during the processing of a less-dominant language, but not the reverse (Linck et al., 2012; van Hell & Dijkstra, 2002). Alternatively, we may find bidirectional effects, where less-dominant languages are co-activated while processing a more-dominant language (Blumenfeld & Marian, 2007; Marian & Spivey, 2003b). In addition to overall effects of language dominance on co-activation, we may find that dominance also modulates the relationship between co-activation and individual differences in language proficiency and cognitive control (Duñabeitia et al., 2010; Green & Abutalebi, 2013).

RQ3 **How do individual differences in language proficiency and cognitive control affect co-activation?**

In their BLINCS model, Shook and Marian (2013) suggest that the magnitude of co-activation will be affected by factors such as proficiency in the target and competitor languages and cognitive control. Predictions about the impact of language proficiency are not straightforward, given mixed findings from bilingual studies. In line with the most-commonly found results, we may see that higher proficiency in a competitor language will be associated with increased co-activation of more-dominant languages (Blumenfeld & Marian, 2007; Blumenfeld & Marian, 2013; Soto & Schmid, 2024), and perhaps of less-dominant languages, as well (Bice & Kroll, 2015; Lee et al., 2018; Marian & Spivey, 2003b). Alternatively, in line with Berghoff and Bylund (2024), we may find that higher proficiency in the competitor language leads to *less* co-activation.

Next, we consider individuals’ proficiency in the target language. While understudied, findings regarding the role of target language proficiency in co-activation point to two possible outcomes. First, higher proficiency in the target language may lead to less cross-linguistic co-activation (Berghoff et al., 2021; Sarrett et al., 2022). Alternatively, we may not find effects of target language proficiency on the co-activation of cross-linguistic competitors (Chambers & Cooke, 2009).

For cognitive control, because most findings from bilingualism research suggest that individuals with stronger cognitive control experience less co-activation (Chen et al., 2017; Giezen et al., 2015; Mercier et al., 2014), we expect a similar pattern in our trilingual context and predict that increased cognitive control will be associated with reduced cross-linguistic competition.

## Methods

The materials for this study, including experimental stimuli, the full data files, and the analysis code, are available at the following link: https://osf.io/7k9bv/.

### Participants

Forty-eight trilingual speakers of HL-Russian, SL-Hebrew, and L3-English, living in Israel, took part in the study. Participants (28 females, 20 males) ranged in age from 19-39 (average age: 27.5) and had acquired SL-Hebrew at an average age of 1.06, but no later than age 5. Seventeen of the participants had been born in the former Soviet Union, while the rest had been born in Israel. All participants began acquiring English in third grade, per the onset of English education in the Israeli school system (Weissblav, 2017). Self-reported proficiency rankings, as well as scores on an objective proficiency assessment (discussed further in Section 2.4), confirmed that participants were most dominant in the main societal language SL-Hebrew (88%, SD = 5%) followed by L3-English (76%, SD = 9%) and HL-Russian (58%, SD = 15%). Participants’ target recognition accuracy on the three eye-tracking blocks was at or near ceiling level. Participant information is summarized in Table 1.

**Table 1.** Participant demographics and language performance. Values represent means with SDs in parentheses, and asterisks (***) indicate significant differences at the *p* < 0.001 level

***N***	48		
**Mean age**	27.3 (4.9)		
**Age range**	19–39		
**Gender**	28 F, 20 M		
	**HL-Russian**	**SL-Hebrew**	**L3-English**
**Mean age of acquisition**	0	1.04 (1.6)	8-9
**Mean self-rating score** 1–5	3.26 (0.85)***	4.82 (0.39)***	4.16 (0.68)***
**Self-rating score range**	1–5	4–5	3–5
**Mean MINT score (%)**	58 (15)***	88 (5)***	76 (9)***
**MINT score range (%)**	13–85	74–99	57–94
**Accurate target recognition rate (%)**	96.2 (19)	99.5 (7)	99.3 (8)

### Eye-tracking

A trilingual visual world paradigm was used to present displays consisting of four black-and-white line drawings (taken from the Akinina et al. (2015) image database). Each four-item display included (a) the target item, (b) a filler item, and (c–d) either filler items or cross-linguistic competitors. All stimuli—whether target, competitor, or filler items—were common concrete nouns.

The experiment contained one block for each language: Russian, Hebrew, and English. At the start of each block, participants completed two practice trials to ensure they understood the task. Following the practice trials, each language block included 40 experimental trials, with 10 from each of four conditions. In the first condition, the four-item display contained the target item and three fillers, with no cross-linguistic competition. In the second and third conditions, the display included the target item, two filler items, and one cross-linguistic competitor (from each of the non-target languages, respectively, per condition). In the fourth condition, the display included the target item, one filler item, and two cross-linguistic competitors—one from each of the non-target languages.

**Figure 2.**
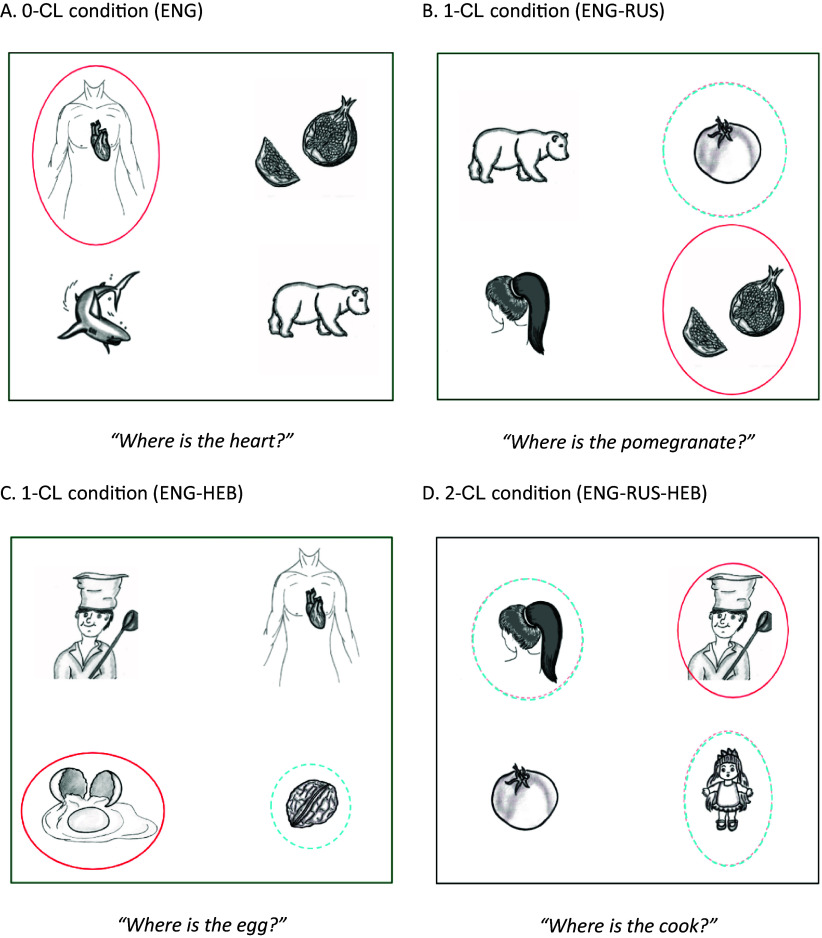
Figure 2 long description. Sample trials for each condition in the English block. In panel A, there are no cross-linguistic competitors (0-CL). In panel B, the English target *pomegranate* competes with the Russian cross-linguistic competitor *pomidor* (tomato.R). In panel C, the English target *egg* competes with the Hebrew *egoz* (nut.H), and in panel D, the English target *cook* competes with Russian *kukla* (doll.R) and Hebrew *kuku* (ponytail.H). Note that the colored ovals in the figure mark the target (red) and competitor items (blue) and did not appear during the experiment. The figure serves illustrative purposes only; images were not repeated in consecutive slides or with the same cohort. *Note*: CL = competitor language.

Figure 2 shows an example set of four slides from the trilingual visual world paradigm, with one slide from each condition. Trials from the four conditions were presented in a random order throughout each language block. However, target–competitor sets were reused and reversed between blocks, such that the target *pomegranate* and competitor *pomidor* (tomato.R) from the English block in Figure 2. B were repeated as target *pomidor* (tomato.R) and competitor *pomegranate* in the Russian block in the English competition condition. The filler items between the two trials were not identical, however.

Participants were seated approximately 45 cm from the 17″ testing screen. At the start of each block, eye fixations were calibrated by the EyeLink using a nine-point calibration routine, at a 1000 Hz sampling rate. Next, participants would see a blank screen with a cross in the center and would need to focus their gaze on it and press the space bar to start a trial. Upon pressing the space bar, participants heard a recorded prompt such as “Where is the **dog**?” and needed to click the relevant image out of the four items in the display. Upon clicking an image, participants would be transferred to another blank screen, where they would refocus on the center cross and press the space bar to begin the next trial. The audio prompts were played immediately upon key press, with no delay, and participants received no feedback on their responses throughout the experiment. All prompts were recorded in a professional recording studio by a balanced trilingual speaker of Russian, Hebrew, and English.

### MINT assessment

To assess participants’ proficiency in each of their three languages, we administered the Multilingual Naming Test, or MINT (Gollan et al., 2012). The MINT was conceived from the start as a vocabulary assessment for multilinguals, distinguishing it from other common picture-naming assessments such as the Boston Naming Test (Kaplan et al., 2001). The MINT includes black-and-white line drawings prompting 68 picture names increasing in difficulty (e.g., “bed” on the lower end and “anvil” on the higher end). Over the last decade, the MINT has become a prevalent lexical proficiency assessment in multilingualism research (Luk & Bialystok, 2013; Marian & Hayakawa, 2021).

Group-level results on the MINT echoed self-ratings and confirmed that the participants in the present study were dominant in their SL-Hebrew, followed by L3-English and HL-Russian, with the greatest score variance in the latter (see Table 1). Separate linear regression analyses on the MINT scores and the self-ratings, with Language as a fixed effect and Participant as a random effect, showed a significant effect of Language. Follow-up pairwise comparisons with Tukey corrections confirmed significant differences (*p* < 0.001) between all three languages on both proficiency assessments.

### Flanker task

To approximate domain-general, non-linguistic cognitive control, participants completed the Flanker task (Botvinick et al., 1999; Eriksen & Eriksen, 1974). In each trial of this task, participants saw five arrows presented in one line and were instructed to press the left or right SHIFT key on the keyboard based on whether the central arrow was pointing left or right. In some trials, all arrows on the screen pointed in the same direction (congruent trials), while in others, the central arrow pointed in the opposite direction from the flanking arrows (incongruent trials). It is expected that incongruent trials result in conflict between the correct and incorrect key-press response, leading to longer reaction times and lower accuracy. Scores on the Flanker task were calculated per participant as the difference between the mean reaction time on incongruent trials and the mean reaction time on congruent trials, reported in milliseconds. A higher score represents greater latencies for incongruent trials, and thus weaker cognitive control, while a lower score represents fewer discrepancies between congruent and incongruent trials, and thus stronger cognitive control. Participants in the present study reached a mean score of 50.5 ms (SD: 28.1 ms), with a range from –7.7 to 133.4. This indicates that some participants were faster on incongruent trials than on congruent trials, while others experienced greater conflict cost.

### Procedure

The present study was approved by the Institutional Review Board at Bar Ilan University. Participants first completed a background questionnaire. Next, following a brief few-minute conversation in Russian with the researchers, participants completed the Russian MINT assessment, eye-tracking calibration, and the Russian eye-tracking block. The sequence of conversational priming, vocabulary assessment, calibration, and eye-tracking block was then repeated consecutively for Hebrew and English. Language testing order was kept consistent across participants in order to parallel their order of acquisition. Finally, participants completed the Flanker task. The full in-person procedure took approximately 1 hour, and at the end of the session, participants were compensated monetarily for their participation.

## Results

### Competitor co-activation

We first compared the mean proportion of looks to the competitor items in each competition condition to the mean proportion of looks to filler items in the control condition^4^. We focused on the time frame of 250 to 1000 ms post-critical word onset, to account for the time it takes for lexical information to influence eye movements (Snedeker & Trueswell, 2004). In the SL-Hebrew block, in conditions with a sole Russian competitor or both a Russian and an English competitor, participants looked to competitors more than to fillers (*p* < 0.0001). However, in the condition with a single English competitor, there were fewer looks to competitors than to fillers (*p* = 0.001), indicating that these competitors were not co-activated. For the models investigating co-activation, see Appendix A.

### Proportion of looks to the competitor item

We next examined competitor activation in each language block by the number of competitor languages present (1, 2) and by language (Russian, Hebrew, English). Below, we report overall proportions of looks to the competitor, followed by the effects of language proficiency and cognitive control on co-activation in each language block. The collected data were analyzed through a linear mixed-effects regression modeled by the formula:

$$\begin{array}{rl} & lmer(looks\_to\_competitor∼competitor\_language^{*}CL\_number^{*}competitor\_MINT\;+\\ & competitor\_language^{*}CL\_number^{*}target\_MINT\;+\\ & competitor\_language^{*}CL\_number^{*}cognitive\_control\;+\\ & (1|Participant)+(1|TRIAL\_INDEX)). \end{array}$$

Our dependent variable was the proportion of looks to a competitor item out of total looks to all items on the display between 250 and 1000 ms after the critical word onset. For fixed effects, we considered interactions between the language of the competitor item, the number of co-activated competitor languages, and language proficiency (MINT) in the competitor and target languages, as well as with cognitive control (Flanker task). We included participants and trial items as random effects. This model was repeated for each language block separately. The full model output can be found in Appendix B.

**Figure 3.**
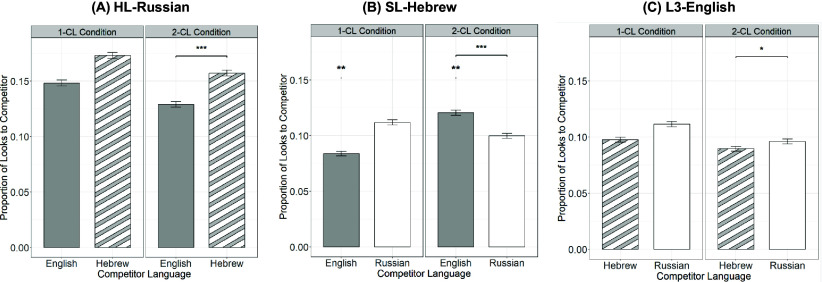
Proportion of looks to the competitor item in each language block ((A) HL-Russian, (B) SL-Hebrew, and (C) L3-English), by the language of the competitor item and by the number of competitor languages. In all language blocks, when two competitor languages were activated at a time, one of them drew significantly more looks than the other. In the Hebrew language block, participants looked to the English competitor more in the 2-CL condition than in the 1-CL condition. Significant differences are marked with * for *p* < 0.05, ** for *p* < 0.01, and *** for *p* < 0.001. *Note*: CL = competitor language.

In each language block, we found that both the number of activated languages and the language of the competitor influenced competitor co-activation (Figure 3, see Appendix C for the full list of pairwise comparisons). Specifically, when processing two competitor languages, participants looked significantly more to one cross-linguistic competitor than the other across all three language blocks (*p* = 0.048 in the L3-English block and *p* < 0.0001 in the other two). In the SL-Hebrew block, the English competitor was activated significantly more in the presence of a Russian competitor than when it was the only competitor (*p* = 0.002). Together, these results suggest that processing two competitor languages at once is qualitatively different than processing one, underscoring a key difference between bilingual and trilingual co-activation. In our main analysis, we consider how the number and type of co-activated languages, in concert with proficiency in the competitor and target languages and with cognitive control, affect the magnitude of co-activation.

Significant three-way interactions were found between the number of competitor languages, the language of the competitor item, and competitor language proficiency for the HL-Russian and SL-Hebrew blocks (*p* < 0.001 for both), as well as with target language proficiency for the SL-Hebrew and L3-English blocks (*p* < 0.001 for both). A simple effect of target language proficiency (*p =* 0.011) was observed in the HL-Russian block, but no interaction effects were found. Additionally, the model showed interactions with cognitive control in both the HL-Russian and the SL-Hebrew language blocks *(p* = 0.003 and *p* = 0.006, respectively). In what follows, we explore the significant three-way interactions between the number of competitor languages, the language of the competitor, and each of our two variables of interest (language proficiency and cognitive control). No significant interactions were observed for competitor language proficiency or cognitive control in the L3-English block, or for target language proficiency in the HL-Russian block, so no follow-up post hoc analyses were conducted for these interactions.

### Language proficiency

**Figure 4.**
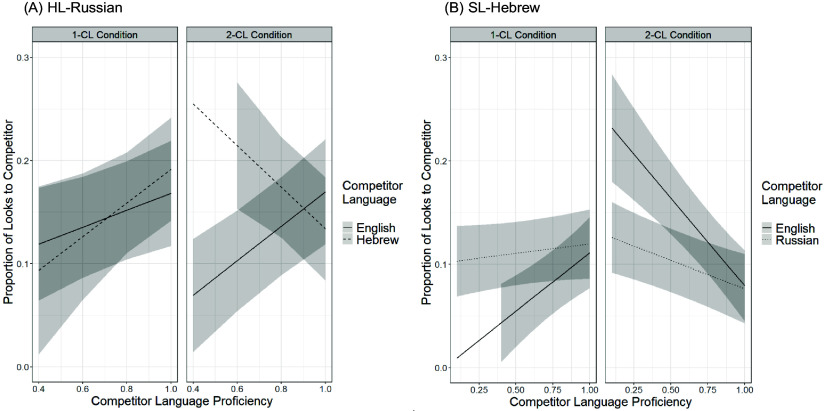
Effect of competitor language proficiency on the proportion of looks to the competitor item in each language block ((A) HL-Russian, (B) SL-Hebrew), by number of competitor languages and by competitor language. In both language blocks, when only one competitor language was present, higher proficiency in the competitor language increased co-activation. When two competitor languages were activated at once, higher proficiency increased co-activation only of the English competitor during Russian processing, while Hebrew co-activation in this condition decreased. Likewise, when processing in dominant Hebrew, higher proficiency in both simultaneously activated competitor languages decreased co-activation. *Note*: CL = competitor language.

In the HL-Russian block, a significant interaction was found between the number of competitor languages, the language of the competitor item, and proficiency in the competitor language as indexed by MINT score (Figure 4A). When only one cross-linguistic competitor was present, higher proficiency in the competitor language predicted more looks to the competing item. When both English and Hebrew competitors were present at the same time, the same effect was observed for English, with higher English proficiency correlating with more looks to the English competitor. The effect was reversed for Hebrew, however, where higher Hebrew proficiency was not associated with more looks to the Hebrew competitor when an English competitor was also present. As the only negative trend, the slope for Hebrew in the latter condition differed significantly from both the slope for English in the same condition and from the slope for Hebrew when it competed alone. Meanwhile, the strength of co-activation did not differ significantly among the remaining competitor languages.

In the dominant SL-Hebrew block (Figure 4B), when there was only one cross-linguistic competitor, higher competitor proficiency predicted more looks to the competitor. However, when both an English and a Russian competitor were present, higher competitor proficiency predicted *fewer* competitor looks for both languages, with a significantly steeper negative slope for the English than the Russian competitor (*p* = 0.006). Curiously, because the target language in this block was the participants’ dominant one, we might have expected to find no effect of competitor language proficiency, rather than a negative one.

We next examined proficiency in the target language. In the HL-Russian block, we found no interaction effects with the number of competitor languages or the language of the competitor. Rather, there was only a simple effect for all conditions, showing that higher target language proficiency predicted fewer looks to all cross-linguistic competitors. No follow-up analyses were conducted.

**Figure 5.**
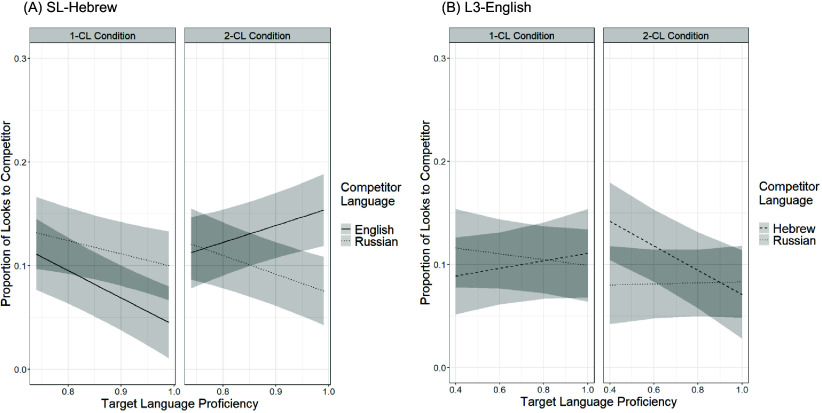
Figure 5 long description. Effect of target language proficiency on the proportion of looks to the competitor item in each language block ((A) SL-Hebrew, (B) L3-English), by number of competitor languages and by competitor language. Increased proficiency in target Hebrew consistently yielded decreased co-activation of HL-Russian, while L3-English activation increased in the presence of a Russian competitor. Increased proficiency in target L3-English increased co-activation of Hebrew and decreased co-activation of Russian when each was present alone and reversed this pattern when both were activated together. *Note*: CL = competitor language.

While processing dominant SL-Hebrew, when only one competitor language was present, higher proficiency in the target language was associated with fewer looks to competing items, and magnitude did not differ by language (see Figure 5A). The same effect was observed for Russian competitors when both Russian and English competitor items were present together. However, surprisingly, higher proficiency in target Hebrew was associated with *more* looks to the English competitor when a Russian competitor was also present.

Finally, in the L3-English block (Figure 5B), when only one competitor language was present, dominant SL-Hebrew competitors were co-activated slightly more with increased target proficiency, and less-dominant HL-Russian competitors slightly less, but the slope difference was not significant. By contrast, when both Russian and Hebrew competitors were present at the same time, Hebrew activation sharply decreased with higher target English proficiency, diverging significantly from the slope of Russian activation (*p* = 0.005). Target proficiency did not significantly modulate looks to the less-dominant Russian competitor whether it was co-activated alone or together with a Hebrew competitor. However, the slope for dominant Hebrew when activated in tandem significantly diverged from its slope when activated alone (*p* = 0.0004).

### Cognitive control

**Figure 6.**
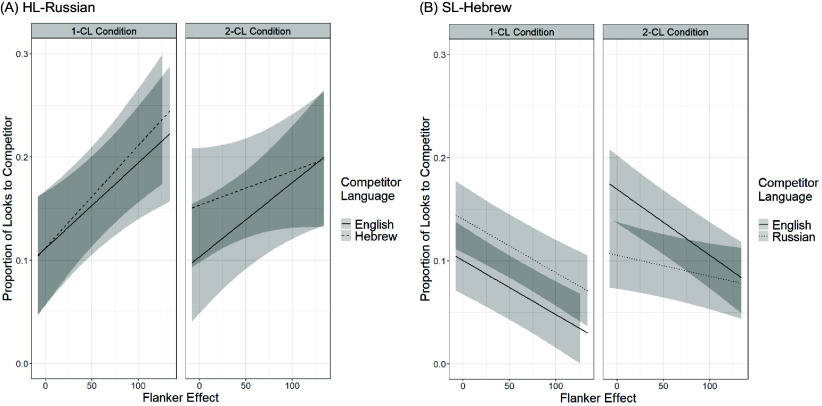
Effect of cognitive control on the proportion of looks to the competitor item in each language block ((A) HL-Russian, (B) SL-Hebrew), by number of competitor languages and by competitor language. Note that higher values indicate weaker cognitive control. Weaker cognitive control yielded more co-activation of both competitor languages in the HL-Russian block and less co-activation of both competitor languages in the SL-Hebrew block. *Note*: CL = competitor language.

In the HL-Russian block, weaker cognitive control—indexed by a larger Flanker effect value—consistently predicted more looks to competitor items, with the magnitude of this relationship varying by the number of competing languages and by the language of the competitor (Figure 6A). Contrasts from the emtrends() simple slopes analysis found that individuals with weaker cognitive control looked significantly more to the Hebrew competitor when it was the sole competitor than when an English competitor was also present (*p* < 0.0001). Furthermore, weaker cognitive control was associated with significantly higher activation of English than Hebrew when both competitor languages were present (*p* = 0.016).

In the SL-Hebrew block, contrasting the Russian and expected results, weaker cognitive control was associated with *fewer* looks to the competitor, regardless of the number of competitor languages or the languages themselves (Figure 6B). Notably, both competitor languages were less dominant than the target language in this block. Thus, the inverse effect of cognitive control may not be so surprising, as co-activation effects may be smaller (or absent, as in the case of the condition with a single English competitor). Follow-up slope analyses showed that weaker cognitive control was more strongly associated with decreased co-activation when Russian was the sole competitor than when it competed alongside English (*p* = 0.022). Additionally, when both Russian and English competitors were present together, weaker cognitive control yielded significantly lower co-activation of the English than the Russian competitor (*p* = 0.001).

## Discussion

The present study examined trilinguals’ co-activation of three languages while processing a visual scene. Consistent with prior models of bilingual processing (Shook & Marian, 2013), we found that trilingual co-activation varies based on the number of simultaneously activated languages, trilinguals’ language dominance in target and competitor languages, and individual differences.

We found that effects of individual differences emerged as a function of the target language. Co-activation varied with competitor language proficiency and cognitive control when participants processed speech in HL-Russian and SL-Hebrew, but not in L3-English. Target proficiency in SL-Hebrew and L3-English showed divergent effects on co-activation based on the number and type of competitor languages, but no such variation was found when processing HL-Russian.

When two competitor languages were activated at the same time, one of them drew significantly more looks than the other, suggesting a co-activation trade-off for simultaneously activated languages. When processing SL-Hebrew, participants only co-activated English competitors that were presented together with Russian ones. This finding further underscores how processing patterns change depending on the languages, and number of languages, activated.

When interpreting the impact of individual differences in proficiency and cognitive control on co-activation, we first focus on the activation of one competitor language, as this context is most comparable to previous work with bilinguals. We then consider the results from two simultaneously activated competitor languages, highlighting novel contributions of our study to understanding processing of multiple competing languages.

### Co-activation of one competitor language

Higher proficiency in any competitor language consistently led to more co-activation of that language. This finding supports both of our proposed hypotheses from RQ3 regarding competitor language proficiency, showing that proficiency in both more-dominant (Blumenfeld & Marian, 2007; Blumenfeld & Marian, 2013; Soto & Schmid, 2024) and less-dominant (Bice & Kroll, 2015; Lee et al., 2018; Marian & Spivey, 2003b) competitor languages could lead to increased co-activation.

Higher proficiency in the target language was often—but not always—associated with decreased competitor co-activation. Echoing findings from Berghoff et al. (2021) and Sarrett et al. (2022), participants more proficient in target SL-Hebrew demonstrated less co-activation of less-dominant Russian. Notably, in the above studies, the competitor language was more dominant than the target, while in the present study this dynamic was reversed, yet yielded the same results, suggesting a robust effect.

In contrast, higher proficiency in target L3-English affected Hebrew and Russian competitors differently, lending credence to our hypothesis that co-activation may vary based on the specific languages at hand (RQ2). We suggest two possible explanations for the divergence: first, SL-Hebrew is activated more due to its relative dominance compared to target L3-English, and second, because the Hebrew block was completed before the English one, activation levels for Hebrew may have been higher. Counterbalancing the order of language testing in future research will help tease apart these possible explanations.

Overall, results from both competitor and target language proficiency demonstrate that, for trilinguals, becoming more proficient in one language may reshape co-activation from the other two languages. Thus, increasing proficiency in one language does not just affect processing in *that* language, but instead appears to impact interactions across trilinguals’ entire language network.

The effects of proficiency on co-activation depended on the relative dominance of target and competitor languages. Likewise, language dominance also modulated the relationship between co-activation and cognitive control. Participants with stronger cognitive control showed *less* co-activation when processing a less-dominant language and *more* co-activation when processing a more-dominant language. Findings from the HL-Russian block echoed results from Mercier et al. (2013) and Chen et al. (2017), who also found that participants with stronger cognitive control experienced less co-activation from more-dominant competitors. These results support our hypothesis from RQ3 that stronger cognitive control may decrease co-activation. However, contrary to the hypotheses posed in RQ2 regarding the effects of competitor language, the magnitude of the effect did not differ based on the language of the competitor, pointing to an overarching mechanism, rather than a language-dependent one.

When processing SL-Hebrew, stronger cognitive control predicted *more* co-activation of less-dominant Russian^5^. These results are aligned with Blumenfeld and Marian (2013), who found a positive relationship between cross-linguistic co-activation and stronger cognitive control in the early stages of word recognition. Our findings support the hypothesis in RQ2, which proposes that the relationship between co-activation and cognitive control may vary based on the target language. Furthermore, our results align with Green and Abutalebi’s (2013) proposal of adaptive control mechanisms and with predictions from Shook and Marian (2013), which suggest an asymmetrical impact of cognitive control as a function of language dominance.

However, in addition to possible top-down explanations of our findings, a bottom-up activation account may also shed light on the results. In particular, the order in which languages were tested may have raised the baseline activation of HL-Russian to become higher than it would be otherwise, such that individuals would activate Russian more, supporting a bottom-up activation account. Alternatively, because participants completed the Hebrew block after the Russian block, our findings may be the result of “negative priming” (Meuter & Allport, 1999). That is, the effort needed to manage more-dominant languages while processing in HL-Russian may have made it more difficult to subsequently control *less*-dominant languages during SL-Hebrew processing. Our findings suggest a possible reversed dominance effect in the trilingual context, which has previously only been reported in bilingual processing (Gavino et al., 2025; Goldrick & Gollan, 2023). Ultimately, the finding that stronger cognitive control predicted more co-activation of less-dominant languages warrants further investigation. Future studies should further examine how sequentially activating three languages impacts language control and activation during spoken word recognition.

### Co-activation of two competitor languages

When two competitors from different languages were presented together, one typically received a greater proportion of looks than the other, regardless of target language. This preference could not be consistently attributed to the more dominant of the two competitor languages, or to relative dominance compared to the target language, suggesting that, in the face of multiple competing languages, the trilingual system prioritizes one.

The relationship between co-activation and proficiency differed when processing two competitor languages, rather than just one. This finding supports our hypotheses from RQ1 and RQ2, wherein both the number of competing languages and the languages themselves modulate the effects of proficiency in trilingual processing. When processing HL-Russian, higher proficiency in competitor Hebrew led to sharply decreased co-activation in the presence of an L3-English competitor. This is surprising as Hebrew was participants’ dominant language, so we may have expected it to increase co-activation with higher proficiency, as it had when competing alone. One possible explanation may be that high-proficiency speakers can more easily resolve Hebrew competition in the presence of increased noise (in this case, other competition), perhaps as a consequence of more experience with competition resolution due to an earlier age of acquisition, as suggested by Schroeder and Marian (2017). It is not clear, however, why the co-activation of L3-English would increase with greater proficiency regardless of the number of simultaneously activated competitor languages.

When processing in SL-Hebrew, co-activation of both competitor languages decreased with higher competitor proficiency. Our findings echo Berghoff and Bylund (2024), who suggested that higher proficiency in a less-dominant language would yield less co-activation of that language. Notably, this effect was observed only when activating two competitor languages together, supporting Schroeder and Marian’s (2017) proposal that activation may be limited in the face of increased competition, or alternatively suggesting that our measure was not sufficiently sensitive to index lower levels of simultaneous co-activation.

Increased target Hebrew proficiency impacted the activation of Russian competitors consistently, regardless of the number of competing languages. Meanwhile, the activation of English competitors sharply increased with higher target Hebrew proficiency when accompanied by Russian competitors. This finding contradicts our hypothesis from RQ3 regarding target language proficiency, which we expected would decrease co-activation.

The result is even more unusual considering that target Hebrew was more dominant than English, such that English activation may have been less likely (Linck et al., 2012; van Hell & Dijkstra, 2002). These complexities could be further explored by examining the relationship between the proficiencies of the two languages. Thus, we echo calls from Duñabeitia et al. (2010) for future work to delve into relative language proficiency as a key factor shaping language processing in order to understand how this variable, in conjunction with other language experience factors such as frequency, recency, and context of use (i.e., Chen et al., 2017), work in tandem to facilitate or impede co-activation.

Finally, higher target L3-English proficiency decreased Hebrew activation when processing two competitor languages, contrasting the trend from Hebrew-only activation. These results align with our hypothesis from RQ3 on the role of target language proficiency (in line with Berghoff et al. (2021) and Sarrett et al. (2022)) but do not shed light on why these same results were not replicated when competitor Hebrew was activated alone.

The divergent effects of proficiency when processing two competitor languages point to joint influence from the number of competing languages (RQ1) and from trilinguals’ dominance in each language (RQ2). The exact combination of target and competitor languages may also impact processing, as different proficiency levels in two more-dominant languages, for example, may have differential effects on co-activation. The mixed findings described above indicate that trilinguals’ language experience idiosyncratically influences how each language is processed or co-activated.

By contrast, we found that cognitive control affected co-activation similarly within each language block regardless of the number of activated languages. As it had in the presence of only one competitor language, stronger cognitive control led to less co-activation when processing in HL-Russian and more co-activation when processing in SL-Hebrew. This consistency in patterns presents cognitive control as a more domain-general capacity that regulates language processing independently of the type and number of competing languages (Schroeder & Marian, 2017).

Interestingly, when processing in HL-Russian, the L3-English competitor was activated more than the simultaneously presented SL-Hebrew competitor, suggesting that this interaction is not a straightforward effect of dominance (whereby we would have expected stronger activation of Hebrew), but perhaps one of language acquisition order. To further investigate effects of dominance and order of acquisition, future studies could assess sequential trilinguals whose third language is also their weakest.

### General discussion

Our examination of the impact of individual differences on trilingual processing yielded four key insights. First, increasing proficiency in one language affects activation patterns of the full linguistic network, not just that one language; and second, these patterns fluctuate based on the exact constellation of languages involved. Third, trilinguals with stronger cognitive control show less co-activation of more-dominant languages and more co-activation of less-dominant languages, demonstrating that effects of cognitive control vary with language dominance. Finally, the relationships between co-activation and individual differences are differentially modulated by the number of simultaneously co-activated languages, suggesting that the addition of a third language inherently changes processing compared to a bilingual system. Thus, we show that not only do trilinguals co-activate all their languages, as had previously been demonstrated for bilinguals, but that activation patterns of each language are in constant flux as a function of language experience and cognitive factors.

Given the nuanced interrelationships between the number of languages activated, language dominance, proficiency, and cognitive control, it is clear that existing models may need to be adapted or expanded to account for the more complex nature of co-activation in multilingual contexts. Future research should work toward a model that incorporates individual differences, dominance dynamics, and the number of activated languages, to build a more comprehensive understanding of how multiple languages interact within a trilingual mind.

### Limitations and future directions

The present study demonstrates, for the first time, how individual differences in trilingual processing can be studied through eye-tracking methodologies. However, we acknowledge a set of limitations in our study that could be refined in future work. First, the stimuli in the present study were controlled only for phonological overlap, and future studies should also account for other characteristics, such as lexical and bigram frequencies. The audio prompts were recorded by a fluent trilingual speaker of all three target languages—future studies could recruit monolingual speakers of each language, as even minor vocal cues may reveal the speaker’s multilingual status and influence co-activation (i.e., Lee & Sidtis, 2017). Future research should also counterbalance the order of languages tested. Finally, explicitly assessing knowledge of competitor labels in all three languages would strengthen the assumption that increased looks to competitors resulted from phonological overlap and co-activation. Likewise, future studies should consider more comprehensive objective and subjective proficiency assessments, to account for linguistic competencies beyond lexical knowledge.

In the present study, we demonstrated that trilingual processing varies as a function of both the number and the types of co-activated languages, suggesting that processing two competitor languages is both quantitatively and qualitatively distinct from processing only one. Although patterns of co-activation when competitor items were present from one non-target language aligned with prior bilingual findings, a direct comparison of trilinguals and bilinguals is still needed to more clearly understand how trilingual and bilingual co-activation and competition resolution differ when the target and competitor languages, proficiencies, dominance, and cognitive control are the same in the two groups.

Finally, while this is a first step in using eye-tracking to test language co-activation and interaction in a trilingual network during natural language processing, future research can more rapidly test and model trilingual and multilingual processing and co-activation in artificial language systems. Future research—with both natural and artificial languages—will need to include populations of multilinguals whose languages overlap or diverge in different ways and whose linguistic (proficiency, dominance, and experience) and cognitive (executive function and memory) profiles vary, for a more complete and accurate understanding of how cognitive systems accommodate multiple languages simultaneously.

## Conclusion

In the present study, we examined the co-activation and interaction of multiple languages in trilinguals. Trilinguals’ processing of one target language and one competitor language was similar to patterns observed with bilinguals. Trilinguals’ processing of one target language and two competitor languages suggests that managing simultaneous co-activation from multiple competitor languages differs from managing one competing language only. Individual differences in language proficiency, dominance, and cognitive control influence the thresholds of activation of each language in the trilingual language network. These findings advance our understanding of how the brain accommodates multiple languages and highlight the interactive nature of multilingual language processing.

## Notes

1 In line with Grosjean (2020), we define language knowledge here as being able to produce meaningful utterances or possessing at least one partial linguistic competence (reading, writing, and speaking understanding).

2 As heritage speakers, our participants include both simultaneous and early sequential bilinguals. To avoid speculating on whether Hebrew would be their 2L1 or their L2, we use the accepted terminology “societal language (SL)” to more accurately capture participants’ language dynamics. Meanwhile, as the third language acquired chronologically, we refer to English as participants’ L3 (Geiss et al., 2022; Siemund, 2023).

3 Fridman and Meir (2025) considered the proportion of looks to the *target* item between different competition conditions. In the present study, we analyze the proportion of looks to the *competitor* item, add new data from the Flanker task, and focus on the impact of individual differences on co-activation (none of which were investigated in Fridman and Meir (2025)).

4 Within-condition comparisons were not used because looks within a display are interdependent (Ito & Knoeferle, 2023) and, in our design, the unequal number of competitors and fillers across conditions would yield a variable baseline.

5 Because we did not find evidence of co-activation for English in the 1-CL condition, we do not discuss it here.

## Replication package

The materials for this study, including experimental stimuli, the full data files, and the analysis code, are available at the following link: https://osf.io/7k9bv/.

## Appendix A

Models comparing looks to the competitor in the competition conditions to looks to the fillers in the control condition in each language block. All competitor conditions indicate co-activation except the 1-CL English condition in the Hebrew block, where competitors are looked at less than fillers. *P*-values reaching significance (below .05) are marked in bold.

**Appendix A1:** Russian block

Predictors	Estimates	95% CI	*p*
(Intercept)	0.10	0.08–0.11	**<0.001**
Condition [1-CL English]	0.05	0.05–0.06	**<0.001**
Condition [1-CL Hebrew]	0.08	0.07–0.08	**<0.001**
Condition [2-CL English & Hebrew]	0.05	0.04–0.05	**<0.001**

**Appendix A2:** Hebrew block

Predictors	Estimates	95% CI	*p*
(Intercept)	0.09	0.08–0.10	**<0.001**
Condition [1-CL English]	−0.01	−0.01 – −0.00	**0.001**
Condition [1-CL Russian]	0.02	0.01–0.02	**<0.001**
Condition [2-CL English & Russian]	0.02	0.01–0.02	**<0.001**

**Appendix A3:** English block

Predictors	Estimates	95% CI	*p*
(Intercept)	0.07	0.06–0.09	**<0.001**
Condition [1-CL Hebrew]	0.02	0.02–0.03	**<0.001**
Condition [1-CL Russian]	0.04	0.03–0.04	**<0.001**
Condition [2-CL Hebrew & Russian]	0.02	0.01–0.02	**<0.001**

**Appendix B:** Mixed-effects model for the three language blocks. *P*-values reaching significance (below .05) are marked in bold.

	Russian		Hebrew		English	
	Estimates, CI	*p*	Estimates, CI	*p*	Estimates, CI	*p*
CL	−0.54, −0.82 – −0.27	**<0.001**	−0.20, −0.47–0.06	0.138	0.22, 0.01–0.13	**0.041**
CL number	−0.07, −0.16–0.02	0.120	−0.12, −0.24 – −0.01	**0.041**	0.14, .03–0.25	**0.016**
Inhibitory control	0.00, −0.00–0.00	**0.005**	0.00, −0.00–.00	**0.027**	0.00, −0.00–0.00	0.578
CL proficiency	−0.00, −0.14–0.13	0.962	0.40, 0.27–0.52	**<0.001**	0.01, −0.21–0.24	0.902
TL proficiency	−0.16, −0.27 – −0.04	**0.011**	−0.69, −0.93 – −0.45	**<0.001**	0.19, 0.07–0.32	**0.003**
CL × CL number	0.45, 0.29–0.61	**<0.001**	0.18, 0.02–0.35	**0.029**	−0.15, −0.27 – −0.02	**0.023**
CL × inhibitory control	0.00, −0.00–0.00	**0.015**	0.00, −0.00–0.00	0.087	0.00, −0.00–0.00	0.864
CL number × inhibitory control	1.00, −0.00–0.00	0.405	0.00, −0.00–0.00	0.297	0.00, −0.00–0.00	0.351
CL × CL proficiency	0.52, 0.27–0.80	**<0.001**	−0.30, −0.45– −0.16	**<0.001**	0.00, –0.23–0.24	0.973
CL number × CL proficiency	0.09, .01–.17	**0.037**	−0.28, −0.36– −0.20	**<0.001**	−0.04, −0.18–0.10	0.573
CL × TL proficiency	0.10, −0.01–0.21	.063	0.62, 0.31–0.92	**<0.001**	−0.25, −0.41– 0.09	**0.002**
CL number × TL proficiency	−0.01, −0.06–0.04	0.694	0.43, 0.28–0.57	**<0.001**	−0.16, −0.23 – −0.08	**<0.001**
CL × CL number × inhibitory control	0.00, −0.00–0.00	**0.003**	0.00, −0.00–0.00	**0.006**	0.00, −0.00–0.00	0.345
CL × CL number × CL proficiency	−0.45, −0.61 – −0.29	**<0.001**	0.21, 0.12–0.30	**<0.001**	−0.01, −0.16–0.13	0.839
CL × CL number × TL proficiency	−0.06, −0.13–0.01	0.081	−0.48, −0.67 – −0.29	**<0.001**	0.19, 0.09−0.29	**<0.001**

*Note.* CL = competitor language; TL = target language.

## Appendix C

Pairwise comparisons for the effects of competitor language and number of competitor languages on the proportion of looks to the competitor, as shown in Figure 3. *P*-values reaching significance (below .05) are marked in bold.

**Appendix C1:** Contrasts by competitor language within the 1- and 2-CL conditions

Language block	Number of CLs	Contrast	Estimate	SE	*z*-Ratio	*p*-Value
HL-Russian	1	English-Hebrew	−0.0087	0.0332	−0.263	0.7922
HL-Russian	2	English-Hebrew	−0.0304	0.0069	−4.380	**<0.0001**
SL-Hebrew	1	English-Russian	−0.0403	0.0207	−1.949	0.0513
SL-Hebrew	2	English-Russian	0.0414	0.0047	8.870	**<0.0001**
L3-English	1	Hebrew-Russian	−0.004	0.0228	−0.175	0.861
L3-English	2	Hebrew-Russian	0.018	0.0091	1.975	**0.048**

**Appendix C2:** Contrasts by number of competitor languages within competitor languages

Language block	Competitor language	Contrast	Estimate	SE	*z*-Ratio	*p*-Value
HL-Russian	English	1- vs. 2-CL condition	0.0137	0.0328	0.419	0.675
HL-Russian	Hebrew	1- vs. 2-CL condition	−0.0079	0.0330	−0.239	0.8110
SL-Hebrew	English	1- vs. 2-CL condition	−0.0631	0.0207	−3.045	**0.0023**
SL-Hebrew	Russian	1- vs. 2-CL condition	0.0186	0.0205	0.910	0.3627
L3-English	Hebrew	1- vs. 2-CL condition	0.002	0.0238	0.084	0.9329
L3-English	Russian	1- vs. 2-CL condition	0.024	0.0213	1.128	0.2595

## Figure 1. Long description

Panel A: A whale labeled KIT, a high-heeled shoe labeled KABLUK, and a cloud labeled CLOUD. Panel B: A map labeled KARTA, a shopping cart labeled CART, a pillow labeled KARIT, and a spoon labeled KAF. Panel C: A map labeled KARTA, a shopping cart labeled CART, a pillow labeled KARIT, and a spoon labeled KAF. Panel D: A map labeled KARTA, a shopping cart labeled CART, a pillow labeled KARIT, a spoon labeled KAF, and a ticket labeled KARTIS. Panel E: A map labeled KARTA and a ticket labeled KARTIS.

Navigate back to Figure 1..

## Figure 2. Long description

Panel A: 0-CL condition (ENG). Illustration shows a heart inside a human body, a pomegranate, a slice of watermelon, a unicorn, and a polar bear. The heart is circled in red. Panel B: 1-CL condition (ENG-RUS). Illustration shows a polar bear, a tomato, a pomegranate, a slice of watermelon, and a woman with a ponytail. The tomato is circled in blue, and the pomegranate and watermelon slice are circled in red. Panel C: 1-CL condition (ENG-HEB). Illustration shows a chef, a human body with a heart, two eggs, and a nut. The eggs are circled in red, and the nut is circled in blue. Panel D: 2-CL condition (ENG-RUS-HEB). Illustration shows a woman with a ponytail, a chef, a tomato, and a doll. The ponytail is circled in blue, the chef is circled in red, and the doll is circled in blue.

Navigate back to Figure 2..

## Figure 5. Long description

The image contains two sets of line graphs labeled as Panel A and Panel B. Panel A is titled SL-Hebrew, and Panel B is titled L3-English. Each panel is divided into two conditions: 1-CL Condition and 2-CL Condition. The x-axis represents Target Language Proficiency, and the y-axis represents the Proportion of Looks to Competitor. In Panel A, the competitor languages are English and Russian, while in Panel B, the competitor languages are Hebrew and Russian. Each line graph shows the relationship between target language proficiency and the proportion of looks to the competitor item for each competitor language. In Panel A, increased proficiency in target Hebrew consistently yields decreased co-activation of HL-Russian. In Panel B, L3-English activation increases in the presence of a Russian competitor. Increased proficiency in target L3-English increases co-activation of Hebrew and decreases co-activation of Russian when each is present alone and reverses this pattern when both are activated together.

Navigate back to Figure 5..
